# Fabrication of gentamicin loaded Col-I/HA multilayers modified titanium coatings for prevention of implant infection

**DOI:** 10.3389/fchem.2022.1019332

**Published:** 2022-11-22

**Authors:** Le Ma, Jiajia Zong, Xiaowei Xun, Xiaoming Hu, Zejing Chen, Quanchao Zhang, Mengxia Peng, Botao Song, Haiyong Ao

**Affiliations:** ^1^ Jiangxi Key Laboratory of Nanobiomaterials, School of Materials Science and Engineering, East China Jiaotong University, Nanchang, China; ^2^ Key Laboratory of Synthetic and Natural Functional Molecule of the Ministry of Education, College of Chemistry and Materials Science, Northwest University, Xi’an, Shaanxi, China

**Keywords:** titanium coatings, Col-I/HA multilayers, drug-release, prevention infection, cytocompatibility

## Abstract

In this study, gentamicin loaded collagen I/hyaluronic acid multilayers modified titanium coating (TC-AA(C/H)_6_-G) was fabricated *via* a layer-by-layer (LBL) covalent immobilization method. The drug releasing properties of collagen I/Hyaluronic acid (Col-I/HA) multilayers and the effect of loaded gentamicin on the antibacterial properties and cytocompatibility of modified TC were investigated. The gentamicin release assay indicated that the Col-I/HA multilayers modified TC exhibited agreeable drug-loading amount (537.22 ± 29.66 µg of gentamicin) and controlled-release performance (240 h of sustained release time). TC-AA(C/H)_6_-G revealed satisfactory antibacterial activity and inhibited the colonization and biofilm formation of *S. aureus*. Fortunately, the functions of hMSCs on TC-AA(C/H)_6_-G did not affected by the loaded gentamicin, and TC-AA(C/H)_6_-G could improve the adhesion, proliferation and osteogenic differentiation of cells, as well as TC-AA(C/H)_6_. *In vivo* animal study indicated that TC-AA(C/H)_6_-G could effectively control intramedullary cavity infection caused by *S. aureus* and prevent bone destruction.

## Introduction

Implant infection is one of the most serious complications of orthopedic surgery (Schierholz and Beuth, 2001). An infection rate of 1.5%–2.5% is reported after primary arthroplasty, while in revision surgery the infection rate is 3.2%–5.6% (Montanaro et al., 2011). More seriously, the infection rates of internal fixation in severe closed fractures and in open fractures are 3.6%–8.1% and high to 20%, respectively (Johnson et al., 2007). As well as lengthening the healing time of the wound, postoperative infection could damage the osseointegration and reduce the utilization efficiency of the implant.

The traditional approach to treat post-operative infection is that after removed the implant through a second surgery, emergent debridement is performed with systemic broad-spectrum antibiotics, and then a new material is implanted after the wound healed. Nevertheless, some surrounding normal tissue followed with inflammation could be removed during debridement, which increases the pain of the patient and prolongs the treatment time. More importantly, the systemic application require high doses of antibiotics, which could cause systemic side effects (Tan et al., 2012b). The topical drug sustained release system could overcome the above-mentioned defects well. The antibacterial agent is loaded into the implant, and release slowly in the local wound area to prevent the adhesion and growth of pathogenic bacteria around the implant (Sun et al., 2019; Chen et al., 2021; Li et al., 2022).

The topical sustained drug release system could effectively solve the defects of traditional drug methods with low efficiency and high toxicity. The drug release systems used for surface modification of titanium-based materials include calcium-phosphorus bioactive ceramics and bioactive macromolecules (Wang et al., 2017; Sandomierski et al., 2022). As one of the most widely used drug sustained-release carriers, collagen I (Col-I) has excellent biocompatibility, degradability, weak antigenicity (Liu et al., 2008; Li et al., 2018; Tripathi et al., 2021). Wachol-Drewek et al. (1996) implanted a gentamicin-loaded collagen sponge into abdominal rot, resulting in a high local gentamicin concentration and remission of infection, while the drug concentration in serum was very low. Hyaluronic acid (HA) is another favored drug-loaded bioactive polymer. Csapó et al. (2018) prepared hyaluronic acid-based colloidal drug delivery systems, which may be a potential candidate for controlled drug release of hydrophobic ketoprofen (KP) molecules. The hyaluronic acid-heparin hydrogel prepared by Bhakta et al. (2012) can control the release of active factor bone morphogenetic protein-2 (BMP-2).

In our previous work (Ao et al., 2013), the collagen I/hyaluronic acid multilayer composite film was constructed on the surface of the plasma sprayed porous titanium coating (TC) by using a layer-by-layer (LBL) covalent immobilization method, which improved biological properties of the titanium coating. The obtained multilayer composite film with a certain thickness may have satisfactory drug-loaded release properties. The intention of this study was to investigate the drug-loaded and controlled-release properties of the composite membrane-modified TC and gentamicin was used as the model drug. The antibacterial properties and cytocompatibility of the drug-loaded composite membrane-modified TC were also explored, as well as intramedullary nail infection model.

## Materials and methods

### Materials

Titanium coating on Ti alloy plates (Φ 10 mm × 2 mm) and rods (Φ 1.6 mm × 20 mm) (denoted as TC) were fabricated by vacuum plasma spraying (VPS, F4-VB, Sulzer Metco, Switzerland) (Xue et al., 2005). Simply, after sandblasting, fine titanium powder was sprayed, followed by coarse titanium powder. Col-I from calf bone with 80,000–100,000 Da of molecular weight was obtained from Saining Bioengineering Technology Co., Ltd, Tianjin, China. HA powder with 300,000 molecular weight was purchased from Bloomage Freda Biopharm Co., Ltd, Jinan, China.

### Fabrication of gentamicin loaded Col-I/HA multilayers on titanium coating

**FIGURE 1:**
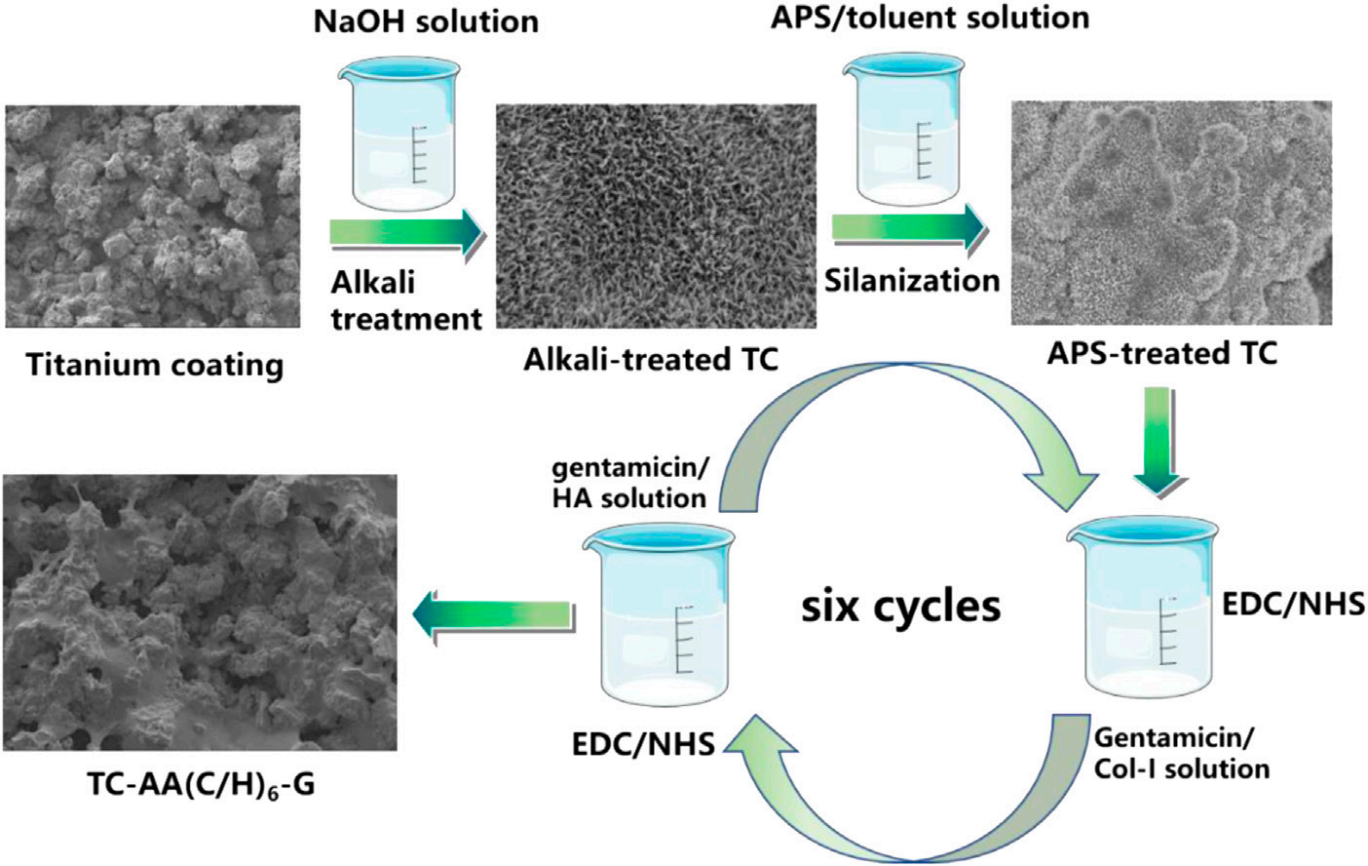
Schematic illustration of the fabrication of gentamicin loaded Col-I/HA multilayers on titanium coating *via* a LBL covalent immobilization technique.

Gentamicin loaded Col-I/HA multilayers modified titanium coating was fabricated by a LBL covalent immobilization technique given by our previous study (Ao et al., 2013). As shown in Figure 1, TC samples were immersed in 5 M NaOH at 80 °C for 12 h, followed by immersing in a boiling aminopropyltriethoxysilane (APS)/toluene solution for 12 h to silanization. Then, the silanized samples were dipped alternately into the gentamicin/Col-I solution (2 mg/ml of gentamicin and 1 mg/ml of Col-I) and gentamicin/HA solution (2 mg/ml of gentamicin and 1 mg/ml of HA) for 30 min, both included 2.5 mg/ml 1-ethyl-3-(3-dimethylaminopropyl) carbodiimide (EDC) and 0.63 mg/ml N-hydroxysuccinimide (NHS). Each dipping process was followed by rinsing with deionized water. After repeating six cycles, the samples were dried under vacuum and denoted as TC-AA(C/H)_6_-G.

Col-I/HA multilayers modified titanium coating (TC-AA(C/H)_6_) as control group was constructed by the LBL covalent immobilization technique. Gentamicin loaded alkali-treated titanium coating (denoted as TC-A-G) and gentamicin loaded Col-I modified titanium coating (denoted as TC-AAC-G) were also prepared to compare the ability of loading and releasing gentamicin. Alkali-treated TCs were immersed into 2 mg/ml gentamicin solution for 24 h at 4°C. As for TC-AAC-G, silanized samples were immersed into the gentamicin/Col-I solution (2 mg/ml of gentamicin and 1 mg/ml of Col-I) for 24 h at 4°C. The solution included 2.5 mg/ml EDC and 0.63 mg/ml NHS. The two obtained samples were both dried under vacuum.

### Release of gentamicin

The release profile of gentamicin was detected according to literature (Popat et al., 2007). One piece of three gentamicin-loaded samples (TC-A-G, TC-AAC-G and TC-AA(C/H)_6_-G) were placed in centrifuge tubes containing 2 ml of phosphate buffered solution (PBS). Centrifuge tubes were placed in a 37°C incubator and shaken at 100 rpm. At time points of 0, 1, 2, 4, 8, 12, 24, 48, 72, 96, 120, 144, 168, 192, 216, and 240 h, all PBS was collected separately, then 2 ml of fresh PBS was added. The PBS collected at each time point was analyzed for cumulative gentamicin release by chemical reaction colorimetry. First, 2.5 g Phthalaldehyde (Sigma), 62.5 ml methanol (Sigma) and 3 ml 2-mercaptoethanol (Sigma) were added to 560 ml sodium borate solution. 50 μl of gentamicin/PBS solution, 50 μl of the reaction solution prepared above and isopropanol was added into a 96-well plate and incubated for 30 min at room temperature. Third, the absorbance value of samples can be measured at 332 nm. Finally, the exact content of gentamicin in PBS was obtained according to the gentamicin standard.

### Zone of inhibition

1 × 10^8^ colony forming unit (CFUs) in 200 μl *S. aureus* (ATCC 25923) suspensions were evenly plated onto tryptone soy agar (TSA), and one specimen of TC-A-G, TC-AAC-G and TC-AA(C/H)_6_-G was placed on the center of the TSA and then cultivated for 24 h at 37°C. The zone of inhibition (ZOI) was observed and their widths were measured as described in the literature (Ao et al., 2019).

### The spread plate method

Three kinds of samples [TC, TC-AA(C/H)_6_ and TC-AA(C/H)_6_-G] were incubated with 1 ml of bacterial suspension containing 1 × 10^6^ CFU/ml for 6 h and 24 h. The bacteria adherent to the samples were collected using ultrasonication at the specific time points. After serial dilution by tenfold, bacteria suspensions were plated onto tryptone soy agar (TSA) for 24 h. The number of bacterial colonies on the substrates were counted and calculated.

### Observation of bacteria by SEM and CLSM

The bacteria/material samples were obtained as stated above. After the samples were fixed in 2.5% glutaraldehyde solution, the bacteria/material specimens were dehydrated using a series of graded ethanol solutions. Then, the samples were observed through a scanning electron microscope (SEM, JEOL JSM-6700F, Japan).

After incubation with three strains for 6 h or 24 h, the materials with colonized bacterial were stained by LIVE/DEAD backlight bacteria viability kits, and then, they were observed with a confocal laser scanning microscope (CLSM, Leica TCS SP2; Leica Micro Systems, Germany).

### Cell attachment and proliferation

The adhesion and proliferation behavior of human mesenchymal stem cells (hMSCs) on the material surface were detected by 3-(4,5-dimethylthiazol-2-yl)-2, 5-diphenyltetrazolium bromide (MTT) assay according to the procedure described in literature (Ao et al., 2014). Among them, the cell concentration of the adhesion experiment was 5×10^4^ cells/well, and the time points were 6, 12, and 24 h. While the cell concentration of the proliferation experiment was 1×10^4^ cells/well, and the time points were 1, 3, and 6 days. In the proliferation assay, the optical density (OD) value at day 1 was measured as a baseline. The proliferation of hMSCs was expressed as the ratio of the OD value relative to the value for day 1 of the same specimen.

### Osteogenic differentiation

Cells were seeded on materials surface at a density of 5×10^4^ cells/well and co-cultured in the osteogenic induction medium for 4, 7 and 14 days. At each defined time point, the alkaline phosphatase activity of the cells on the surface of the material was compared using the quantitative method of pNPP, as described in the literature (Nie et al., 2016). Other cell/material specimens co-cultured in the osteogenic induction medium for 14 days were stained with alkaline phosphatase (ALP) to qualitatively display the osteogenic differentiation of the cells.

After 21 days of co-culture in the osteogenic induction medium, the osteogenic differentiation of cells was also observed by alizarin red staining. Briefly, after fixed with 95% ethanol for 10 min, cell/material specimens were dyed in 0.1% alizarin red solution at 37°C for 45 min, followed by dried and photographed (Nikon D90, Japan). Quantitative analysis of alizarin red staining as follows: The surface of the above-mentioned alizarin red-stained material was dissolved in 10% cetylpyridinum chloride (CPC) (Sigma-Aldrich) in sodium phosphate (pH = 7), and then the absorbance value (OD value) was measured on a microplate reader (Bio-tek, United States) at a wavelength of 620 nm (Yang et al., 2016).

### Surgical procedures

Thirty 5-month-old female Sprague-Dawley rats that were randomly assigned to three groups were used. The use of animals and the experimental protocol were approved by the Animal Experimental Ethics Committee of East China Jiaotong University. After general anesthesia and sterilization, a hole with a diameter of 1.6 mm and depth of 20 mm was drilled through cortical and cancellous bone to access the medullary cavity. After injected 10^4^ CFU of ATCC 25923, three implants (TC, TC-AA(C/H)_6_ and TC-AA(C/H)_6_-G) were inserted into the holes. All animals were tested on the day of surgery (day 0) and on the 1st, 3rd, 5th, 7th, 14th, 28th, 35th, and 42nd day after the operation. Ear temperature was measured by a digital infrared thermometer (TERUMO, Zhejiang, China) and animals were weighed with an electronic balance (TCS, Shanghai, China). Other observed indicators included left knee joint swelling and wound exudation. Animals were euthanized after 6 weeks, and the femurs with the implants were aseptically harvested. Five femurs were dissected longitudinally symmetrically along the midline of the femur and sored according to the scoring methods introduced in literature (Ao et al., 2019).

### Microbiological evaluations

The removed implants were rolled on a TSA plate and incubated at 37°C for 24 h. The bacterial growth morphology of the TSA plate was photographed. On the other hand, the number of bacteria colonized on other removed implants and bones were investigated by the spread plate method. Specially, the femurs of each group were weighed, and then the quantity of bacteria in each bone was expressed relative to the femur weight (CFUs/g).

### Radiographic evaluations

Femur and knee joint lateral radiographs of all groups were obtained on 1st, 21st, and 42th day post-surgery. Radiographic manifestations were assessed with a scoring system previously detailed in the literature (Peng et al., 2015).

Five femurs with implant in each group were randomly chosen. After the femurs were fixed with the 4% v/v buffered formaldehyde, all femurs that were collected were evaluated by micro-CT (SCANCO MEDICAL, μCT 80, CH-8306 Bruttisellen, Switzerland). 3D high-resolution images were obtained from overall, longitudinal and transverse sections. Bone volume/total volume (BV/TV) and the cortical bone mineral density (BMD) of the samples were reconstructed and analyzed.

### Bone histopathology

After fixed in 4% v/v buffered formaldehyde for 48 h and decalcified for 2 weeks using a Rapid Decalcifier (DeCa DX-1000, Pro-Cure Medical Technology Co. Ltd., Hong Kong), medial femur halves were embedded in paraffin and sectioned using a microtome (CUT 6062; SLEE, Medical, Germany) to obtain 5 μm longitudinal and transverse sections. Hematoxylin and eosin (H&E) staining was used to assess morphology, and Giemsa staining was used to assess bacterial contamination. The pathological of bone tissue infection was quantitatively scored by the following indicators, intraosseous acute inflammation (IAI), intraosseous chronic inflammation (ICI), periosteal inflammation (PI) and bone necrosis (BN) (Nie et al., 2017).

### Statistical analysis

Experiments were repeated five times for each sample. Statistical analysis was performed with SPSS software. Results were expressed as the mean ± standard deviation. Statistical significance was determined if *p* < 0.05.

## Results and discussion

### Release of gentamicin

**FIGURE 2:**
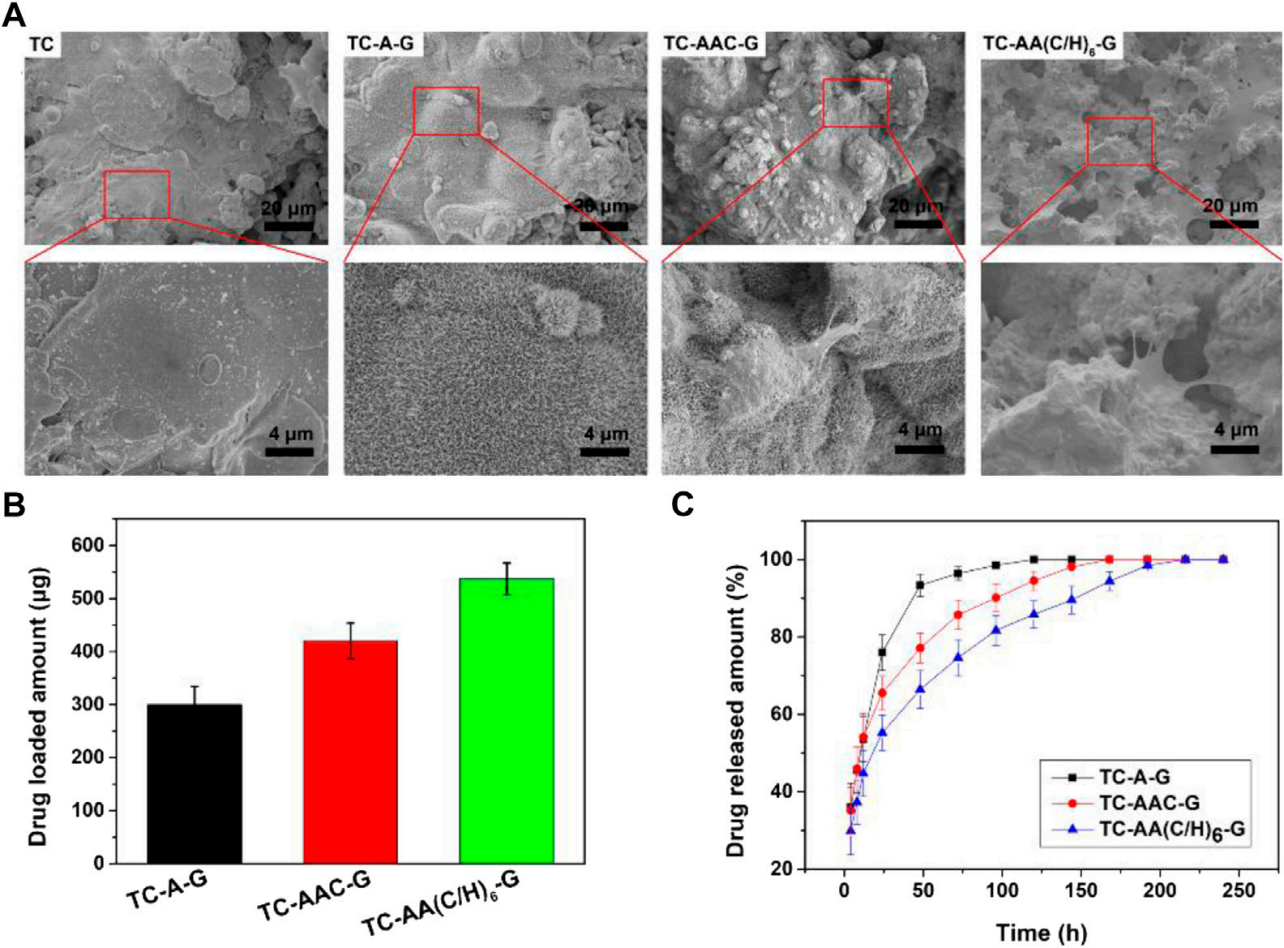
SEM of samples **(A)**, Drug loaded amount **(B)** and cumulative release curves of gentamicin **(C)** of TC-A-G, TC-AAC-G and TC-AA(C/H)_6_-G.

Figure 2 shows the SEM of samples and the drug loaded amount and cumulative release curves of gentamicin of three drug-loaded modified titanium coatings. The gentamicin loaded amount of TC-AA(C/H)_6_-G was 537.22 ± 29.66 µg, higher than that of TC-A-G (299.96 ± 34.66 µg) and TC-AAC-G (420.02 ± 33.86 µg), because of the favourable drug loading properties of Col-I and HA (Figure 2B). It can be seen that burst release occurred in the release curve of TC-A-G, with the vast majority of loaded gentamicin released within 48 h. In contrast, the release time of gentamicin for TC-AAC-G and TC-AA(C/H)_6_-G was longer than that of TC-A-G, up to 168 h and 240 h, respectively (Figure 2C). As shown in our previous study (Ao et al., 2014), after alkali-treated, a microporous structure and abundant of Ti-OH groups were formed on the surface of titanium coating, which could be loaded with drugs. However, the micropores formed by alkali treatment were interconnected and had open porous structure, and do not have a controlled release effect. Many literatures have proved that both type I collagen and hyaluronic acid have excellent drug-loaded release properties (Ray et al., 2013; Ioan et al., 2019; Bayer, 2020). Compared with TC-A-G, TC-AAC-G can load more drugs and has a better sustained release effect due to the grafted collagen. Nevertheless, compared with TC-AAC-G, a composite membrane could be seen on the surface of TC-AA(C/H)_6_-G, which completely covered with porous and spicules structures produced by alkali treatment (Figure 2A). The composite membrane with considerable thickness endowed the TC-AA(C/H)_6_ with decent drug-loading and controlled-release performance, as shown in Figures 2A,B.

### Zone of inhibition

**FIGURE 3:**
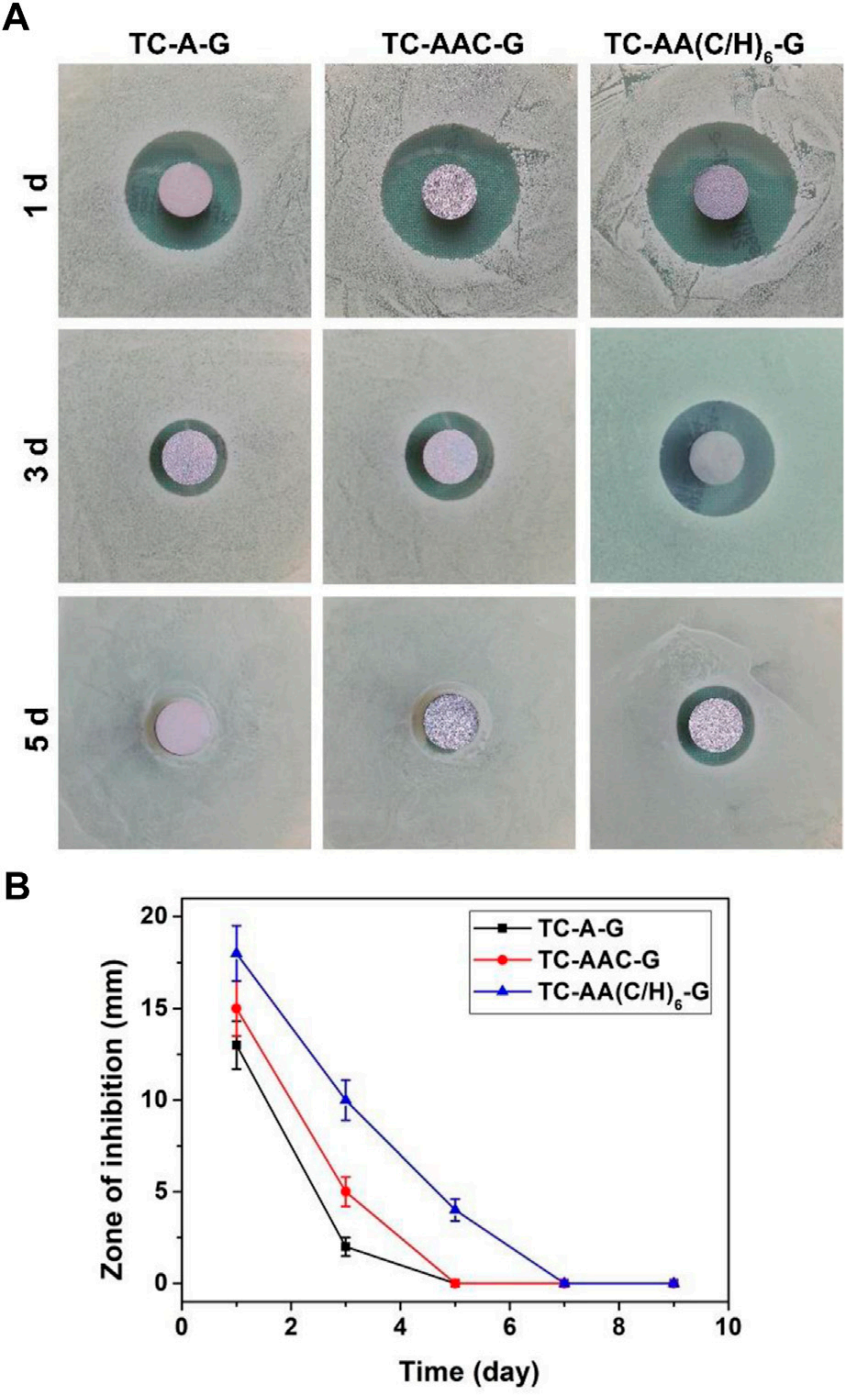
The zone of inhibition (ZOI) of multilayer membrane modified TCs loading gentamycin. **(A)** The ZOI against *S. aureus* produced by different specimens at different time points; **(B)** The ZOI diameters of all specimens.

The zone of inhibition for the three drug-loaded titanium coatings are shown in Figure 3. At the same time point, the ZOI of TC-AA(C/H)_6_-G was larger than that of TC-A-G and TC-AAC-G. The reason might be that TC-AA(C/H)_6_-G could releases more gentamicin per unit time than TC-A-G and TC-AAC-G, which could kill bacteria in a wider range and form the larger bacteriostatic ring. On the other hand, the inhibition rings of TC-A-G and TC-AAC-G disappeared on the 5th day, while the inhibition rings of TC-AA(C/H)_6_-G persisted until the 7th day. This result was because of the better sustained-release properties of TC-AA(C/H)_6_-G, and indicated that TC-AA(C/H)_6_-G had improved sustained antibacterial ability compared with TC-A-G and TC-AAC-G.

### Antibacterial properties

**FIGURE 4:**
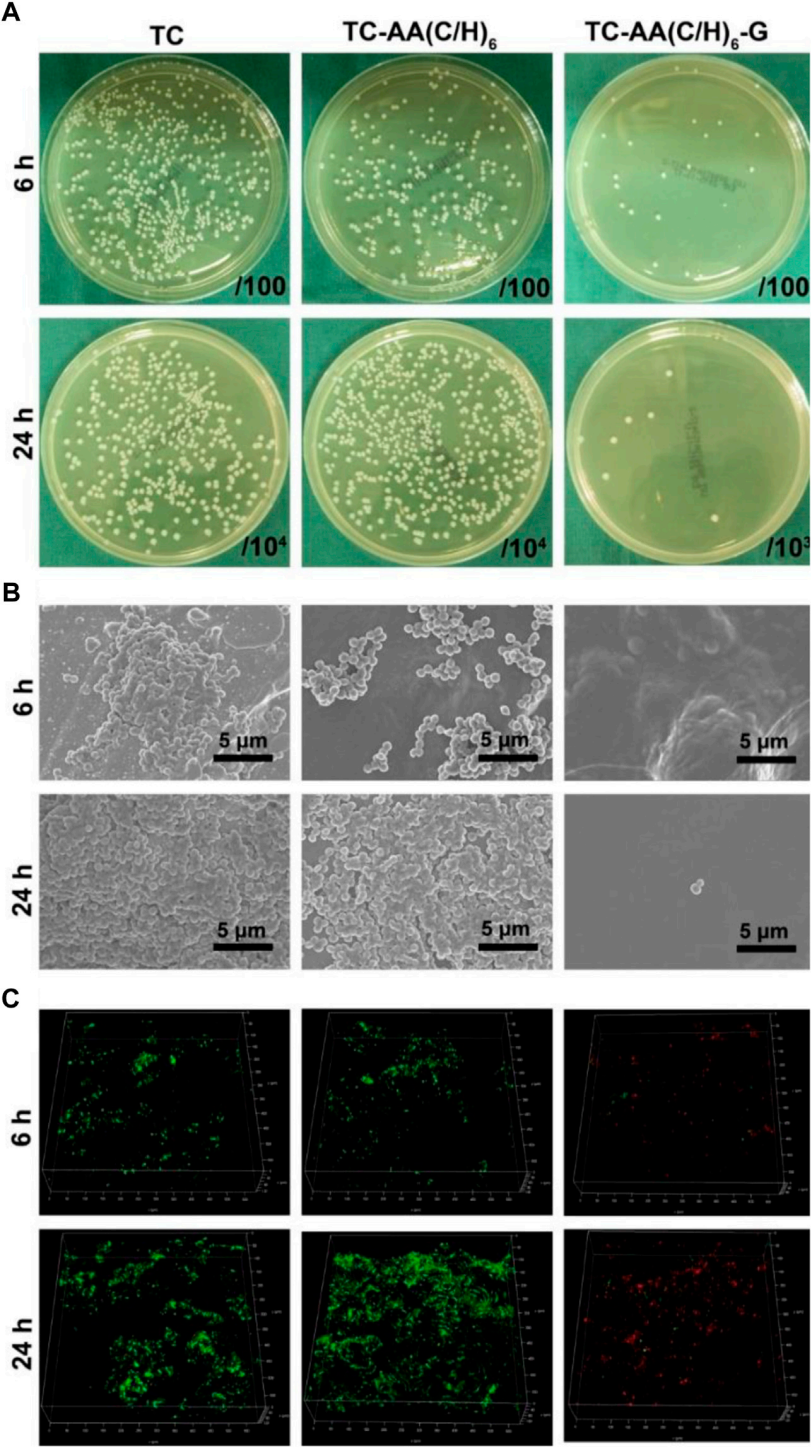
Antibacterial properties of TC-AA(C/H)_6_-G. **(A)** Bacterial colonization and biofilm formation by the spread plate method; **(B)** SEM of bacteria on the three different specimens after incubated 6 h and 24 h; **(C)** The CLSM views of bacterial colonization and biofilm formation on the three kinds of samples, green for living bacteria and red for dead bacteria.

To investigate the bacteriostatic rate of TC-AA(C/H)_6_-G, the spread plate method was employed and the results were shown in Figure 4A. The number of bacteria on the surface of TC-AA(C/H)_6_-G was much less than that of TC and TC-AA(C/H)_6_ at both time points (6 h and 24 h), indicating that the sample TC-AA(C/H)_6_ could inhibit bacterial colonization and biofilm formation. After counting and calculating, the bacteriostatic rate of TC-AA(C/H)_6_-G was 94.3 ± 2.5% at 6 h, and 99.8 ± 0.2% at 24 h. We also noticed that the bacteriostatic rate of TC-AA(C/H)_6_ was also 42.5 ± 3.2% at 6 h (Table 1). This might be because hyaluronic acid has a certain anti-bacterial adhesion ability. Pirnazar et al. (1999) found that hyaluronic acid could inhibit bacterial adhesion and reproduction but had no bactericidal effect. Drago et al. (2014) also reported that hyaluronic acid was able to interfere with bacterial adhesion to a cellular substrate in a concentration dependent manner. After 24 h of incubation, the bacteriostatic rate of TC-AA(C/H)_6_ was only 15.3 ± 2.6%, which meant there was basically no bacteriostatic effect (Table 1).

**TABLE 1:** The bacteriostatic rate of samples compared with TC.

Co-cultured time (h)	TC	TC-AA(C/H)6	TC-AA(C/H)6-G
6	0	42.5 ± 3.2%	94.3 ± 2.5%
24	0	15.3 ± 2.6%	99.8 ± 0.2%

The fabulous antibacterial activities of TC-AA(C/H)_6_-G was further verified by SEM and CLSM. As shown in Figure 4B, the surfaces of TC and TC-AA(C/H)_6_ had colonized a large number of bacteria at 6 h, and a thick biofilm was formed on their surface at 24 h. Bacteria were hardly found on the surface of TC-AA(C/H)_6_-G at both time points. From the view of CLSM, most of bacteria on TC-AA(C/H)_6_-G were dead (red), meaning that bacteria colonized on its surface could be killed by gentamicin. Nearly all bacteria on TC and TC-AA(C/H)_6_ were alive (green) due to the lack of germicidal ability (Figure 4C).

### Cytocompatibility

**FIGURE 5:**
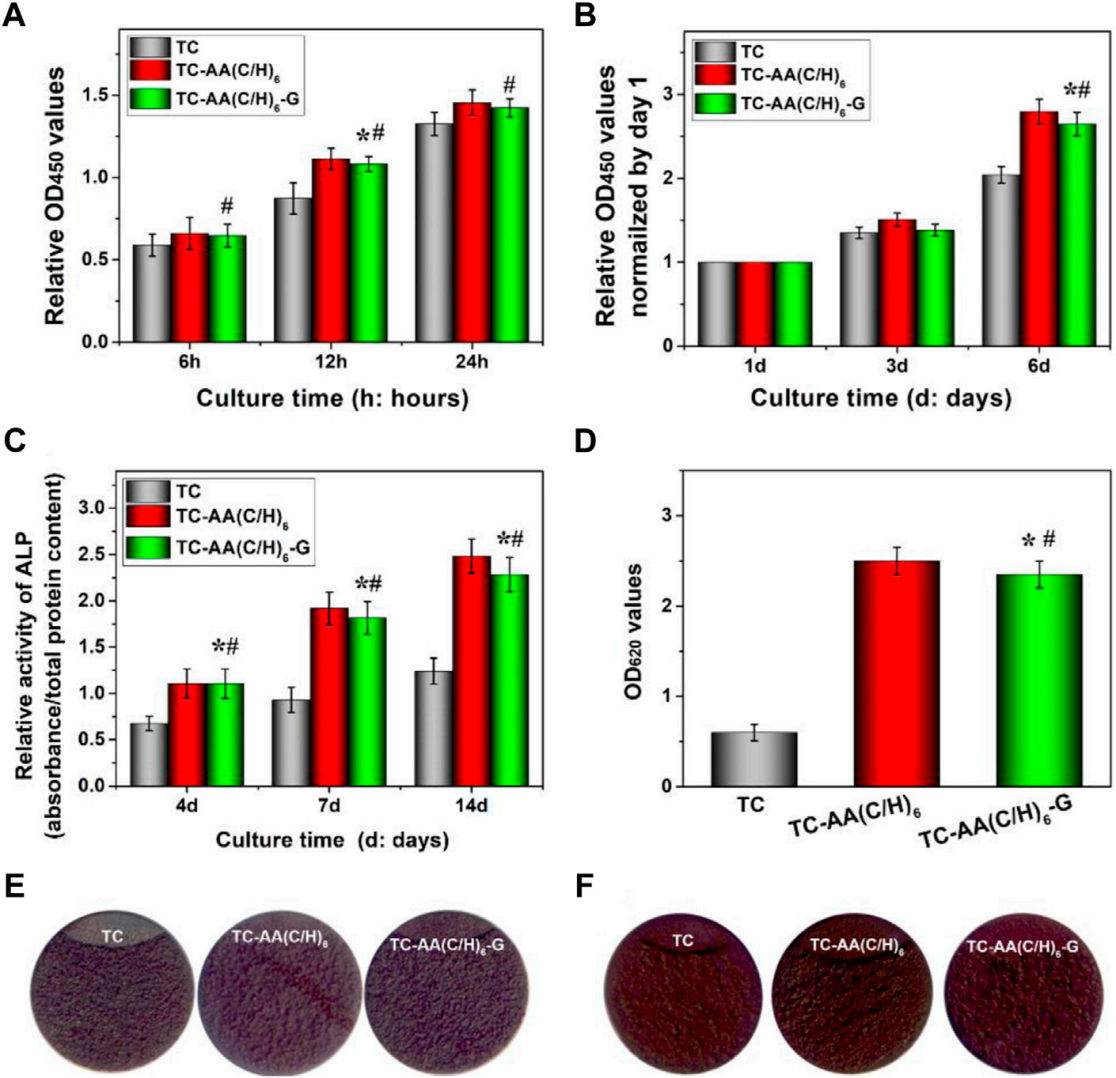
Cytocompatibility of TC, TC-AA(C/H)_6_ and TC-AA(C/H)_6_-G. **(A)** Attachment of hMSCs on three different samples; **(B)** Proliferation of hMSCs on three different samples; **(C)** ALP activity was measured using the pNPP assay and normalized based on the protein content per specimen at days 4, 7 and 14; **(D)** Colorimetric quantitative analysis of the extracellular matrix mineralization on the samples after 21 days of incubation; **(E)** ALP staining was performed at day 14; **(F)** Alizarin red staining. *p < 0.05, there has significant difference between TC-AA(C/H)_6_-G and TC. #*p* > 0.05, there has no significant difference between TC-AA(C/H)_6_-G and TC-AA(C/H)_6_.

Figure 5A shows the adhesion of hMSCs on the surfaces of TC, TC-AA(C/H)_6_ and TC-AA(C/H)_6_-G. The number of hMSCs on TC-AA(C/H)_6_ and TC-AA(C/H)_6_-G was higher than that on TC at all the specified time points. Especially at 12 h, there was a significant difference between TC and the other two. But there is no significant difference between TC-AA(C/H)_6_ and TC-AA(C/H)_6_-G. The results of the adhesion experiment indicated that the Col-I/HA composite membrane constructed on TC, could promote the adhesion of cells, which was not affected by the loaded gentamicin.

Figure 5B shows the proliferation of hMSCs on the surface of TC, TC-AA(C/H)_6_, and TC-AA(C/H)_6_-G. To eliminate the influence of adhesion differences on the proliferation results, the OD value on day 1 was used as the benchmark, and the proliferation rate of other time points relative to the benchmark was used to represent the proliferation rate of cells on samples (Tan et al., 2012a). As can be seen from the results, the proliferation rate of TC-AA(C/H)_6_-G was significantly higher than that of TC, but showed no significant difference compared with that of TC-AA(C/H)_6_. The results indicated that TC-AA(C/H)_6_-G could promote cell proliferation as well as TC-AA(C/H)_6_.

The osteogenic differentiation of hMSCs on different samples had also been investigated. The quantitative determination of ALP activity was shown in Figure 5C. ALP expression of group TC-AA(C/H)_6_ and TC-AA(C/H)_6_-G was significantly higher than TC at all time points. In particular, the ALP expression of group TC-AA(C/H)_6_-G was lower than that of group TC-AA(C/H)_6_ on day 7 and 14, but the difference between them was not statistically significant. The images of ALP staining performed on day 14 further showed the stronger ALP positivity of hMSCs on TC-AA(C/H)_6_ and TC-AA(C/H)_6_-G compared with TC (Figure 5E). On day 21 after osteogenic induction, calcium nodules generated by cells were stained red by alizarin red. Clearly, the calcium nodules on TC-AA(C/H)_6_ and TC-AA(C/H)_6_-G were manifestly more than that on TC (Figures 5D,F). ALP is an enzyme expressed by mesenchymal stem cells during osteogenesis and a well-defined marker for their differentiation, while calcium deposition is critical factors for mineralization of cells and bone-implant interfacial osteointegration (Bai et al., 2020). The results of ALP activity analysis and alizarin red staining demonstrated that TC-AA(C/H)_6_-G could significantly promote the osteogenic differentiation and mineralization of hMSCs.

### The condition of the animals during the experiment

**FIGURE 6:**
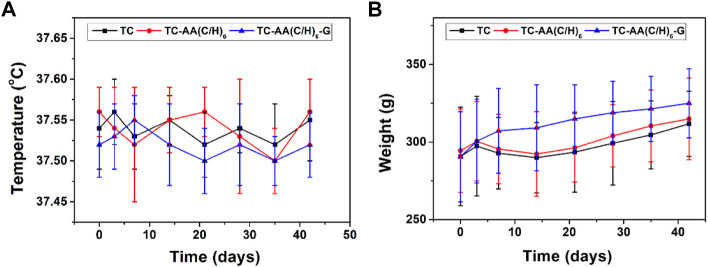
Postoperative changes in temperature **(A)** and weight **(B)** of animals.

Figure 6 showed the changes in body temperature and weight of animals after operation. The body temperature of all animals was between 37.45 and 37.60, which remained normal during the experimental observation period. The animal weight of TC and TC-AA(C/H)_6_ groups increased slightly during the first 3 days, following by decreased and reached the lowest point on the 15th day, which was influenced by the infection. On the other hand, the animal weight of TC-AA(C/H)_6_-G group was not affected by the injected bacteria and increased steadily during the follow-up period. During the follow-up period, no obvious systemic complications occurred in all animals. Most of the animals in the TC and TC-AA(C/H)_6_ groups had swelling symptoms in the left knee joint, and the left lower limb in the TC-AA(C/H)_6_-G experiment was normal.

### Gross appearance of femurs

**FIGURE 7:**
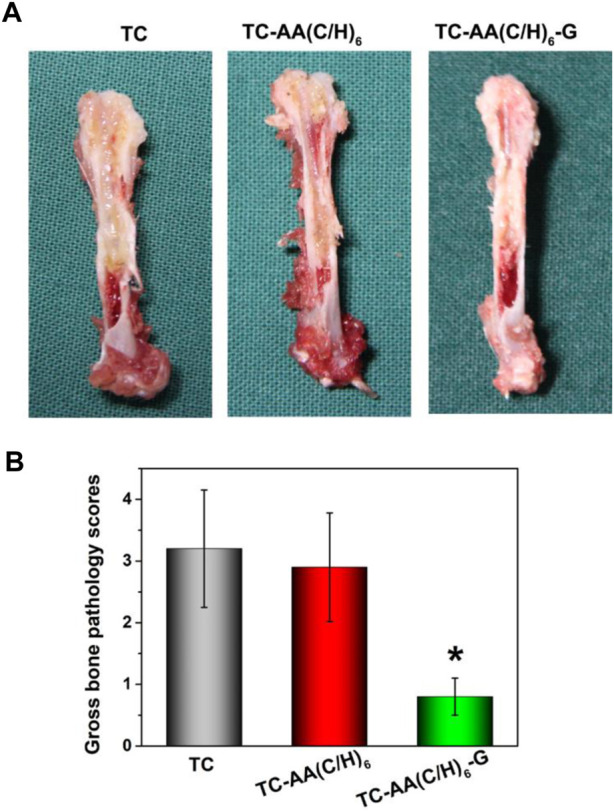
-Gross appearance and scores of longitudinal sections of three kinds of femurs. **(A)** TC and TC-AA(C/H)_6_ groups showed signs of purulent infections, while the TC-AA(C/H)_6_-G group showed no obvious infection signs; **(B)** The score of the TC-AA(C/H)_6_-G group was significantly lower than other two groups, *p < 0.05.

Longitudinal sections of femurs were shown in Figure 7A. The femurs of group TC and TC-AA(C/H)_6_ appeared noticeable purulent infection lesions, including the formation of intramedullary pus, osteolytic lesions and periosteal reaction. While no infection lesion was found in the TC-AA(C/H)_6_-G group. For the gross pathological scores of animal specimens, the scores of TC, TC-AA(C/H)_6_ and TC-AA(C/H)_6_-G were 3.24 ± 0.95, 2.92 ± 0.88, and 0.83 ± 0.51, respectively (Figure 7B). The score of the TC-AA(C/H)_6_-G group was significantly lower than that of the other two groups (*p < 0.05). From the results of gross appearance of femurs, it could be preliminarily concluded that the TC-AA(C/H)_6_-G group inhibited the intramedullary cavity infection caused by *S. aureus*.

### Microbiological evaluation

**FIGURE 8:**
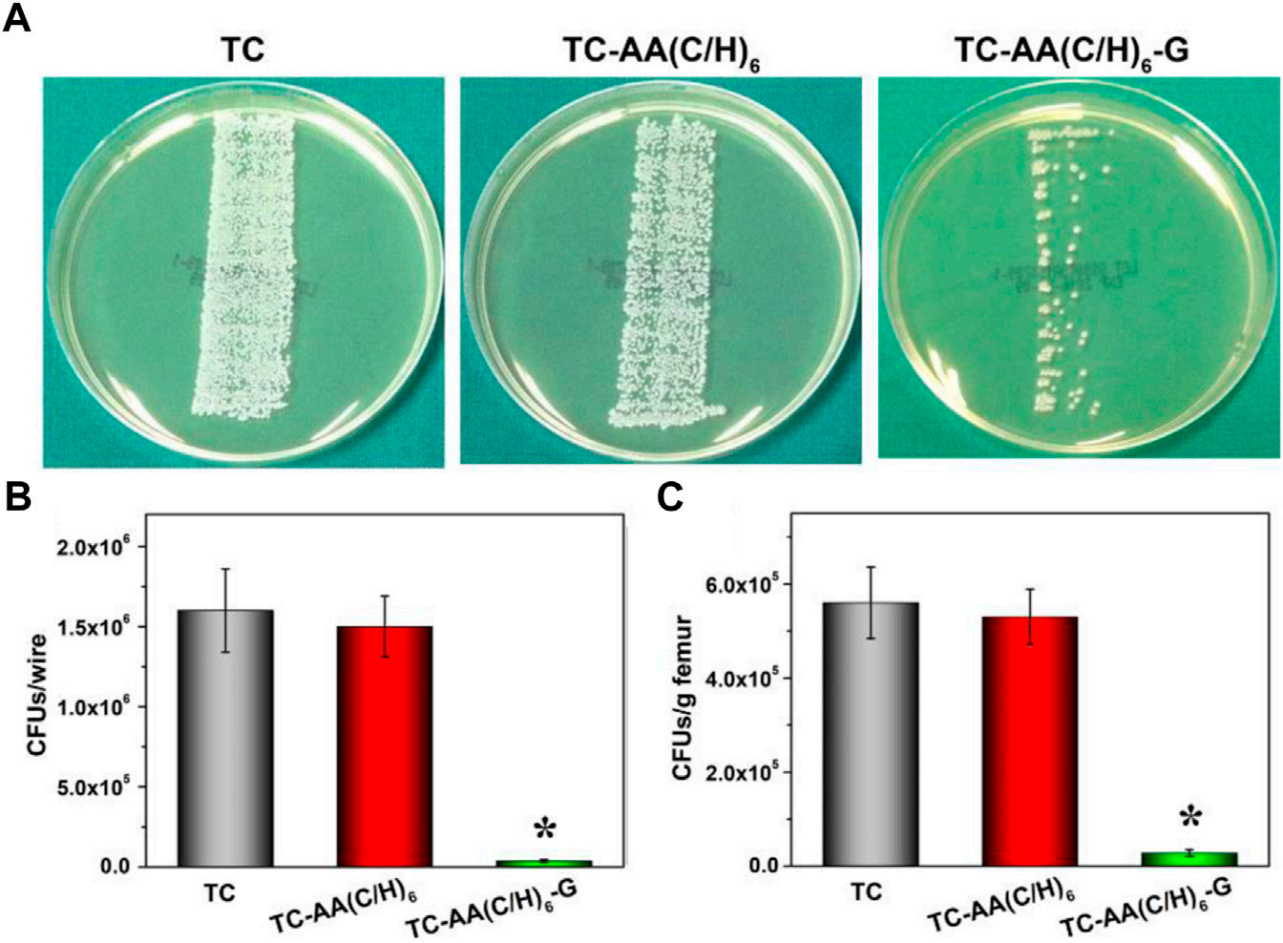
Quantification of bacteria obtained from explanted implants and bone tissue on day of sacrifice. **(A)** Growth of bacterial colonies of implants on TSA; **(B)** The number of CFU per implant; **(C)** Amount of CFU per gram of bone. *p < 0.05 compared with TC-AA(C/H)_6_-G and other two groups.

Figure 8A showed the results of growth of bacterial colonies of implants on TSA. There was a large amount of bacterial colonized on implant TC and TC-AA(C/H)_6_, while only a few bacterial colonies could be found on implant TC-AA(C/H)_6_-G. The results of quantitative analysis also showed that the bacteria on implant TC-AA(C/H)_6_-G (3.6 ± 0.95 × 10^4^ CFU) were evidently less than that on implant TC (1.62 ± 0.26 × 10^6^ CFU) and TC-AA(C/H)_6_ (1.58 ± 0.19 × 10^6^ CFU) (Figure 8B). Coincidentally, the bacterial count of femoral tissue of group TC-AA(C/H)_6_-G was significantly lower than that of group TC and TC-AA(C/H)_6_ (Figure 8C).

### Radiographic evaluation

**FIGURE 9:**
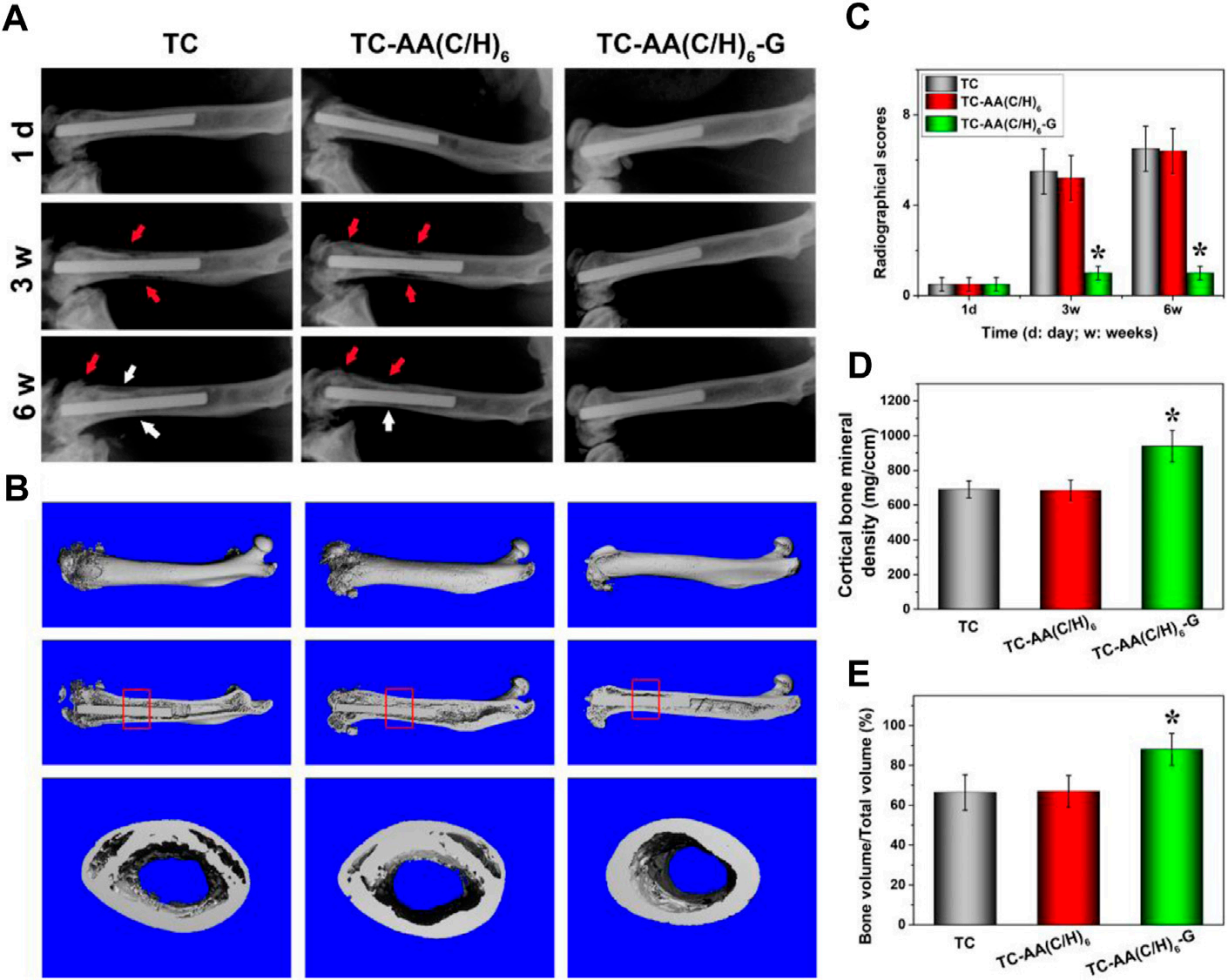
**(A)** X-ray photograph of left femur in lateral view 1 day, 3 and 6 weeks after operation. Typical signs include osteolytic lesions (black arrowhead) and periosteal new bone formation (white arrowhead); **(B)** 3D images of overall, longitudinal and transverse sections; **(C)** Quantitative analysis of the X-ray images; **(D)** the cortical one mineral density (BMD) of ROI; **(E)** bone volume/total volume (BV/TV) of ROI. *p < 0.05 compared with TC-AA(C/H)_6_-G and other two groups.

Figure 9A shows the X-ray imaging analysis at three postoperative time points. As for TC and TC-AA(C/H)_6_ groups, osteolysis and periosteal reactions around the distal femurs were observed obviously after 3 weeks, osteosclerosis and deformities were also evident after 6 weeks post-surgery. Conversely, no obvious imaging signs of bone infection were found in the TC-AA(C/H)_6_-G group at the selected time points. The results of quantitative imaging analysis were shown in Figure 9C. In the TC and TC-AA(C/H)_6_ groups, radiographic scores gradually increased after surgery, whereas in the TC-AA(C/H)_6_-G group, there was no clearly visible improvement. At 3rd week and 6th week, the statistical score of the TC-AA(C/H)_6_-G group was significantly lower than that of the TC and TC-AA(C/H)_6_ groups.

The radiographic observations mentioned above were confirmed by micro-CT analyses. As seen from Figures 9A,B, a significant amount of damage to the compact bone in both TC and TC-AA(C/H)_6_ groups, while the femoral cortical bones were intact in the TC-AA(C/H)_6_-G group. The BMD of the interest region of the TC-AA(C/H)_6_-G group was 939.63 ± 90.52 mg/ccm. Because of infection, the BMD of the interest region of the TC and TC-AA(C/H)_6_ groups decreased to 689.86 ± 55.67 mg/ccm and 683.22 ± 60.48 mg/ccm, respectively (Figure 9D). The bone volume fraction of the interest region in the TC-AA(C/H)_6_-G group was 88.04 ± 8.32%, which was significantly higher than 66.43 ± 9.12% in the TC group and 66.95 ± 8.54% in the TC-AA(C/H)_6_ group (Figure 9E).

### Bone histopathology

**FIGURE 10:**
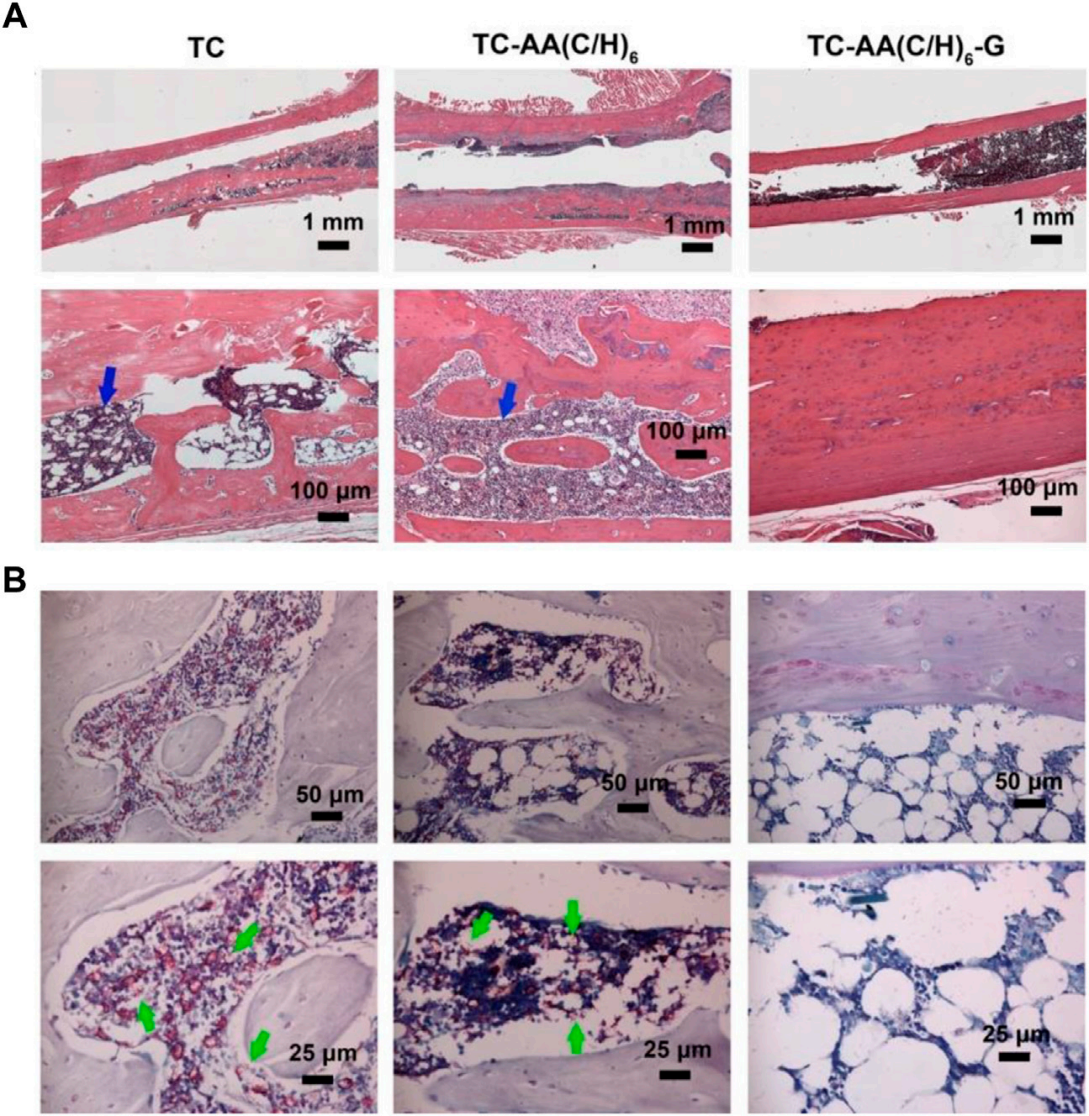
Representative photomicrographs of longitudinal sections of femurs. **(A)** H&E staining; **(B)** Giemsa staining.

The morphological change on the left femur was assessed by H&E staining (Figure 10A), and the bacterial residue was confirmed by Giemsa staining (Figure 9B). In the TC and TC-AA(C/H)_6_ groups, there was obvious destruction of cortical bone, which was accompanied by intracortical abscesses, inflammatory cell infiltration, and medullary sequestrum formation. Many bacteria could be observed within the cavities of damaged cortical bone from the Giemsa staining. Whereas, there was no evident bone destruction only relatively slight inflammatory cell infiltration in the TC-AA(C/H)_6_-G group. Meanwhile, there were few bacteria can be found in the intramedullary cavities of the TC-AA(C/H)_6_-G group (Figure 10B). The results also indicated that the invasive bacteria have been inhibited by gentamicin, as shown in the literature (Nie et al., 2022).

## Conclusion

In summary, gentamicin loaded Col/HA multilayers modified titanium coatings has been prepared by LBL covalent immobilization method. The Col-I/HA multilayer thin film modified on TC exhibited agreeable drug-loading capacity (537.22 ± 29.66 µg) and controlled-release performance compared with TC-A and TC-AAC. The slow release of gentamicin endowed TC-AA(C/H)_6_-G satisfactory and sustained antibacterial properties with inhibition to the colonization and biofilm formation of *S. aureus*, while the Col/HA multilayers improved cytocompatibility of TC-AA(C/H)_6_-G and promoted the adhesion, proliferation and osteogenic differentiation of hMSCs. Furthermore, TC-AA(C/H)_6_-G inhibited intramedullary cavity infection caused by *S. aureus*.

## Data Availability

The raw data supporting the conclusion of this article will be made available by the authors, without undue reservation.
